# Macrophage Activation in the Dorsal Root Ganglion in Rats Developing Autotomy after Peripheral Nerve Injury

**DOI:** 10.3390/ijms222312801

**Published:** 2021-11-26

**Authors:** Xiang Xu, Xijie Zhou, Jian Du, Xiao Liu, Liming Qing, Blake N. Johnson, Xiaofeng Jia

**Affiliations:** 1 Department of Neurosurgery, University of Maryland School of Medicine, Baltimore, MD 21201, USA; Xiang.xu@som.umaryland.edu (X.X.); xzhou@som.umaryland.edu (X.Z.); JDu@som.umaryland.edu (J.D.); xiao.liu@som.umaryland.edu (X.L.); LQing@som.umaryland.edu (L.Q.); 2 Department of Industrial and Systems Engineering, School of Neuroscience, Virginia Tech, Blacksburg, VA 24061, USA; bnj@vt.edu; 3 Department of Biomedical Engineering, Johns Hopkins University School of Medicine, Baltimore, MD 21205, USA; 4 Department of Anesthesiology and Critical Care Medicine, Johns Hopkins University School of Medicine, Baltimore, MD 21205, USA; 5 Department of Orthopedics, University of Maryland School of Medicine, Baltimore, MD 21201, USA; 6 Department of Anatomy and Neurobiology, University of Maryland School of Medicine, Baltimore, MD 21201, USA

**Keywords:** autotomy, peripheral nerve injury, macrophage, DRG, neuropathic pain

## Abstract

Autotomy, self-mutilation of a denervated limb, is common in animals after peripheral nerve injury (PNI) and is a reliable proxy for neuropathic pain in humans. Understanding the occurrence and treatment of autotomy remains challenging. The objective of this study was to investigate the occurrence of autotomy in nude and Wistar rats and evaluate the differences in macrophage activation and fiber sensitization contributing to the understanding of autotomy behavior. Autotomy in nude and Wistar rats was observed and evaluated 6 and 12 weeks after sciatic nerve repair surgery. The numbers of macrophages and the types of neurons in the dorsal root ganglion (DRG) between the two groups were compared by immunofluorescence studies. Immunostaining of T cells in the DRG was also assessed. Nude rats engaged in autotomy with less frequency than Wistar rats. Autotomy symptoms were also relatively less severe in nude rats. Immunofluorescence studies revealed increased macrophage accumulation and activation in the DRG of Wistar rats. The percentage of NF200+ neurons was higher at 6 and 12 weeks in Wistar rats compared to nude rats, but the percentage of CGRP+ neurons did not differ between two groups. Additionally, macrophages were concentrated around NF200-labeled A fibers. At 6 and 12 weeks following PNI, CD4+ T cells were not found in the DRG of the two groups. The accumulation and activation of macrophages in the DRG may account for the increased frequency and severity of autotomy in Wistar rats. Our results also suggest that A fiber neurons in the DRG play an important role in autotomy.

## 1. Introduction

Peripheral nerve injury (PNI) is one of the most common clinical injuries and affects approximately 1 million people worldwide every year [1]. Neuropathic pain, which is a persistent, spontaneous burning or shooting sensation, is often present in patients with neuroma formation and those who underwent neurectomy with sutured repair following PNI [2,3]. Neuropathic pain following PNI seriously impairs the patient’s sleep, mood, and quality of life [4].

Autotomy, self-mutilation of a denervated limb [5,6,7,8], is very common in animals after PNI (e.g., mice, rats, and rabbits), especially in rats after sciatic nerve transection, when the incidence of autotomy reaches as high as 40–80% [5,7,9]. This behavior is painful and affects animal behavioral outcomes. Most studies attribute autotomy behavior to the formation of neuromas [8,10,11]. However, autotomy can also be observed in crush injuries as well as cutting and re-suturing of the sciatic nerve in rats [5]. This is consistent with the presence of neuropathic pain after nerve repair in humans. In general, autotomy in animals is a reliable proxy for neuropathic pain in humans [7,8]. However, there is a limited understanding of the mechanisms that cause autotomy.

Neuropathic pain is a manifestation of damage to a complex neural network and pathway. While a variety of injury mechanisms exist among different models, they complicate pain treatment and often result in unsatisfactory treatment [2,4]. Macrophages in the dorsal root ganglion (DRG) are shown to play an important role in the initiation and maintenance of pain [12]. Previous studies show that some approaches to improve nerve fiber growth, such as administration of brain-derived growth factor (BDGF), have undesirably increased autotomy behavior [8,13]. More research on autotomy is needed to understand and treat neuropathic pain.

In our PNI studies [14,15], we observed that autotomy in nude rats was less common compared to other rat species. However, the reason for the relatively reduced incidence of autotomy in nude rats is unknown. Previous reports showed that a deficiency of T cells in nude rats attenuated their pain hypersensitivity compared to wild rats [16]. However, the difference in autotomy between the two groups has not been reported. The sensitization of primary neurons, a process in which sensory afferents become hyper-responsive to extracellular nociceptive stimuli [17], contributes to neuropathic pain [4,13,18]. Primary neurons can be divided into four categories: Aβ myelinated fibers, Aδ myelinated fibers, peptidergic C fibers, nonpeptidergic C fibers [19]. Many studies show that C fibers are often sensitized and play a key role in neuropathic pain [2,20]. Neuron fibers (Aβ, Aδ, and C fibers) in the DRG play major roles in neuropathic pain depending on the injury [21,22]. Recently, Yu et al. showed that DRG resident macrophages in C57 mice contribute to both the initiation and persistence of neuropathic pain, but infiltrating blood macrophages do not [12]. However, the roles of neurons and macrophages in the DRG and the contribution to the autotomy difference between nude and Wistar rats remain unclear.

Thus, investigating the differences in macrophage activation and fiber sensitization, the sensitization of nociceptive pathways in fiber [23], between nude and Wistar rats, could improve our understanding of autotomy behavior and catalyze new strategies for pain treatment. Here, we evaluate the autotomy difference between nude and Wistar rats and the role of macrophage activation after sciatic nerve transection. Macrophages in the DRG were compared, and the different types of neuron fibers in the DRG were analyzed to elucidate their role in the development of autotomy.

## 2. Results

### 2.1. Nude Rats Exhibit Less Autotomy than Wistar Rats Following Sciatic Nerve Transection

**Figure 1:**
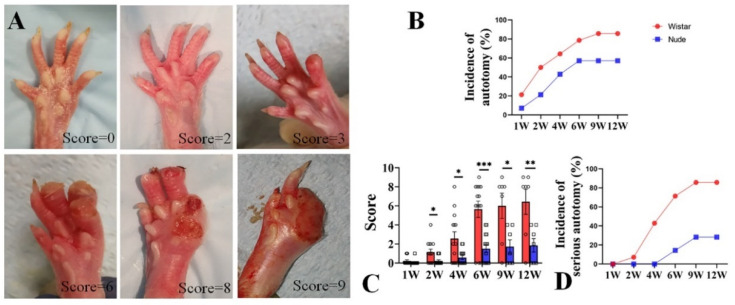
Autotomy in nude and Wistar rats. (**A**). Representative photographs and Wall’s scores of autotomy in the rat after peripheral nerve injury. (**B**). The incidence of autotomy in Wistar rats was higher at different weeks than in nude rats. (**C**). The Wall’s scores of autotomy in Wistar rats were significantly higher than those of nude rats both at different weeks. (**D**). More serious-type autotomy in Wistar rats was observed compared to nude rats. Student’s *t*-test in C. Sample size (n): n = 14 at 1, 2, 4, and 6 weeks and n = 7 at 9 and 12 weeks. * *p* < 0.05, ** *p* < 0.01, *** *p* < 0.001; Wistar rats vs. nude rats.

Autotomy was scored with a modified Wall’s Score (Table 1 and Figure 1A). After surgery, autotomy was observed in Wistar rats within one week, as early as 4 days. At 1 week, the incidence of autotomy was low in both groups (Wistar, 2 of 14, 21.4% vs. nude, 1 of 14, 7.14%). From 2 to 6 weeks, the incidence of autotomy increased rapidly in both groups. While autotomy occurred within the first 6 weeks and lasted throughout the 12-week experiment, new autotomy occurred within the 6–12-week period after surgery. The incidence of autotomy in the Wistar rat group was higher than in the nude rat group. At 2 and 4 weeks, autotomy behavior was observed in half of Wistar rats (7 of 14) and 64.3% Wistar rats (9 of 14). At 6 weeks, 11 of the 14 Wistar rats (78.6%) developed autotomy. At 9 and 12 weeks, both six of the seven Wistar rats (85.7%) exhibited autotomy. The incidence of autotomy was lower in the nude rat group: 3 of 14 nude rats (21.4%), 6 of 14 nude rats (42.9%), 8 of 14 nude rats (57.1%), 4 of 7 nude rats (57.1%), and 4 of 7 nude rats (57.1%) exhibited autotomy at 2, 4, 6, 9, and 12 weeks (Figure 1B), respectively. At the late 6 weeks, the incidence of autotomy was increased by 7.1% in Wistar rats, and there was no increase in nude rats.

Similar to Wistar rats, autotomy in nude rats began in the first week but increased in severity over the course of the 12-week study. At 1 week, the mean autotomy scores between the two groups exhibited no significant difference (0.07 ± 0.26 vs. 0.21 ± 0.42, respectively, *p* > 0.05). However, the mean autotomy scores in nude rats were significantly lower than Wistar rats at late weeks (2 weeks 0.21 ± 0.11 vs. 1.14 ± 0.35, respectively, *p* < 0.05; 4 weeks 0.57 ± 0.20 vs. 2.57 ± 0.70, respectively, *p* < 0.05; 6 weeks 1.50 ± 0.42 vs. 5.64 ± 0.86, respectively, *p* < 0.001; 9 weeks 1.70 ± 0.71 vs. 6.0 ± 1.30, *p* < 0.05; and 12 weeks 1.86 ± 0.71 vs. 6.43 ± 1.32, *p* < 0.01, respectively) (Figure 1C). During the 6–12 week period, the increase in scores was less than that in 0–6 weeks in both groups (nude, 0.36 vs. 1.50, Wistar, 0.79 vs. 5.64). In addition, severe autotomy behavior (score ≥ 4) in nude rats was lower than Wistar rats (0% (0 of 14) vs. 7.1% (1 of 14) at 2 weeks, 0% (0 of 14) vs. 42.9% (6 of 14) at 4 weeks, 14.3% (2 of 14) vs. 71.4% (10 of 14) at 6 weeks, 14.3% (1 of 7) vs. 71.4% (5 of 7) at 9 weeks, and 28.3% (2 of 7) vs. 85.7% (6 of 7) at 12 weeks, respectively) (Figure 1D). Similarly, the emergence of new severe autotomy during the 6–12-week period was less during the late 6-week period in both groups (nude, 14% vs. 14.3%; Wistar, 14.3% vs. 71.4%). These data indicate that autotomy in nude rats was less frequent and severe than in Wistar rats. Although the progression of autotomy was observed during the 6–12-week period, the development of autotomy in both groups was slower and less severe compared to the first six weeks.

### 2.2. Macrophages in DRG Are Activated in Nerve Injury

**Figure 2:**
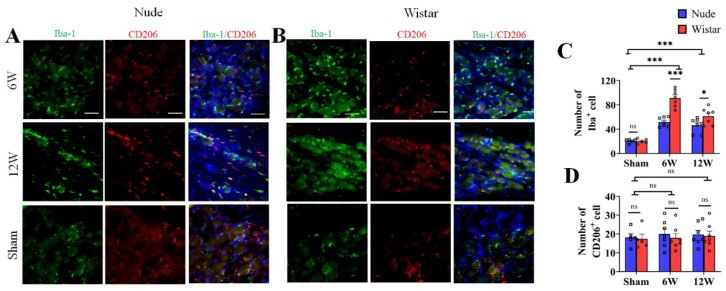
The number of activated (Iba1+) and M2 macrophages (CD206+) in the DRG. (**A**,**B**) Immunofluorescence staining of activated macrophages (Iba1, green) and M2 (CD206, red) in DRG tissue in sham, 6-week, and 12-week groups (**A**,**B**). Following nerve injury, both groups exhibited more activated macrophages than sham rats in DRG tissue; however, M2 macrophages (CD206) exhibited similar expression among all groups. (**C**,**D**). Quantitative analysis associated with the percentage of activated macrophages (**C**) and M2 (**D**). The number of activated macrophages in the DRG of Wistar rats was more than that in nude rats at 6 or 12 weeks. The number of M2 macrophages exhibited no difference. Scale bar 50 μm. Two-way ANOVA with Bonferroni’s post-test was applied. Sample size: n = 7 at 6 or 12 weeks and n = 5 in sham groups (Wistar and nude rats). * *p* < 0.05, *** *p* < 0.001; nude rats vs. Wistar rats, or sham vs. 6 W or 12 W.

Previous studies show that macrophages accumulate in the DRG and are activated after nerve injury [24,25]. To evaluate the potential changes of DRG macrophages associated with the observed differences in autotomy between the two groups, immunofluorescence staining was performed on harvested DRG at 6 and 12 weeks after surgery. Iba1 served as a marker for activated macrophages, and CD206 served as a marker for type 2 macrophages. To explore the role of macrophages in autotomy behavior, the amount of macrophage accumulation between the two groups was detected by immunofluorescence staining. The number of Iba1 marked macrophages in the DRG of Wistar rats at 6 weeks following peripheral nerve transection was increased approximately 4-fold relative to that observed in sham rats (91.14 ± 5.26 vs. 20.00 ± 1.79, *p* < 0.001), while only approximately 2-fold in nude rats (51.71 ± 2.95, *p* < 0.001) (Figure 2A–C). At 12 weeks, more macrophage accumulation was observed in Wistar rats than in nude rats (Figure 2C). Wistar rats exhibited a higher number of Iba1-marked macrophages in the DRG than that in nude rats (61.29 ± 5.17 vs. 46.29 ± 4.46, *p* < 0.05) (Figure 2C). Although the number of Iba1-marked cells remained relatively high in Wistar rats compared to sham rats (61.29 ± 5.17 vs. 20.00 ± 1.79, *p* < 0.001), it decreased significantly in Wistar rats from 6 weeks at 12 weeks (91.14 ± 5.26 vs. 61.29 ± 5.17, *p* < 0.001). However, there was no significant difference in Iba1-labeled macrophage accumulation in the DRG of nude rats between 6 and 12 weeks (51.71 ± 2.95 vs. 46.29 ± 4.46, *p* > 0.05) (Figure 2A–C). The number of CD206-marked cells was similar between the groups and exhibited no significant differences across the various time points of the study. For example, CD206-marked cells in Wistar and nude rats exhibited 17.40 ± 2.50, 18.20 ± 2.08 in sham groups (*p* > 0.05); 17.86 ± 2.38, 20.00 ± 2.73 at 6 weeks (*p* > 0.05); and 18.57 ± 2.64, 19.71 ± 2.24 at week 12 (*p* > 0.05), respectively (Figure 2A,B,D). Negative controls were verified (Supplementary Figure S1). These data show that macrophages accumulated in the DRG after PNI, and there was relatively more activated macrophage accumulation in Wistar than in nude rats. These results suggest that macrophage activation in the DRG may contribute to autotomy.

### 2.3. Myelinated A Fiber Neurons Are Significant Contributors to Autotomy

**Figure 3:**
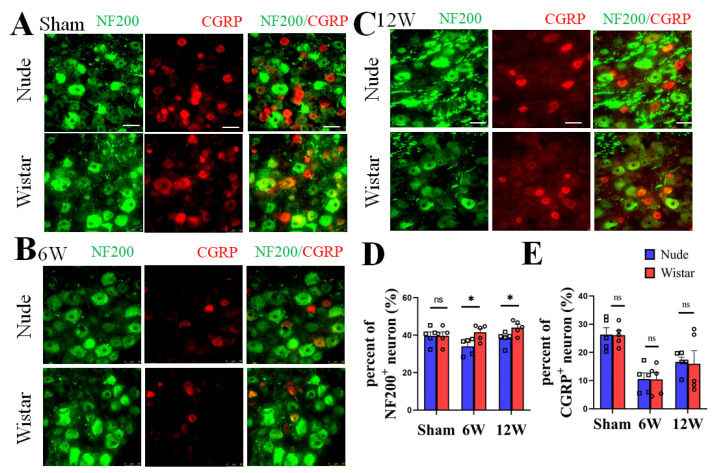
The expression of NF200+ and CGRP+ neurons in DRG. Representative immunofluorescence images of NF200+ and CGRP+ neurons in DRG at sham (**A**), 6 (**B**), and 12 weeks (**C**). (**D**) Quantitative analysis showed the percentage of NF200+ neurons in DRG of Wistar rat was higher than nude rat both at 6 and 12 weeks. There was no difference observed in the percentage of NF200+ neurons in the DRG in the sham groups. (**E**) Quantitative analysis showed that the nude and Wistar rat groups at 6 or 12 weeks, as well as the two sham groups, exhibited no difference in CGRP+ neurons. Scale bar 50 μm. Two-way ANOVA with Bonferroni’s post-test was applied. Sample size: n = 5. * *p* < 0.05; nude rats vs. Wistar rats.

DRG neurons, as recipients and primary neurons in pain pathways, have different fiber types [2]. NF200 has also been reported as a specific marker for A-fibers [26,27]. To verify the type of nerve fibers present following PNI between the two groups, immunofluorescence staining of the neurons in the DRG at 6 and 12 weeks was performed. We found no significant difference in the percentage of NF200+ neurons in the DRG between nude and Wistar rats (39.60% ± 2.11 vs. 39.16% ± 1.77, *p* > 0.05) in sham rats. The percentage of NF200+ neurons in DRG was significantly higher in the Wistar rats compared to nude rats at 6 weeks (41.56% ± 1.89 vs. 34.06% ± 2.00, *p* < 0.05) (Figure 3A,C). A similar result was observed at 12 weeks (44.05% ± 1.65 vs. 38.21% ± 1.67, *p* < 0.05) (Figure 3B,C). There was also no difference in the percentage of CGRP+ neurons between the two sham groups (Wistar rats 26.12% ± 1.68 vs. nude rat 26.32% ± 2.47, *p* > 0.05). However, the percentage of CGRP+ neurons did not significantly differ between the two groups at 6 (Wistar rats 10.43% ± 2.34 vs. nude rat 10.57% ± 2.19, *p* > 0.05) or 12 weeks (Wistar rats 15.94% ± 4.67 vs. nude rat 16.62% ± 3.84, *p* > 0.05). Relative to the fraction at 6 weeks, the percentage of CGRP+ neurons in the DRG increased at 12 weeks in nude and Wistar rat groups (Wistar rats 10.43% ± 2.34 vs. 15.94% ± 4.67, *p* < 0.001; nude rats 10.57% ± 2.19 vs. 16.62% ± 3.84, *p* < 0.001) (Figure 3A,D). Our data show that while the percentage of NF200+ neurons in the DRG of Wistar rats was higher than that in nude rats following PNI, the percentage of CGRP+ neurons was not.

### 2.4. Activated Macrophages Surround Myelinated A Fiber Neurons

**Figure 4:**
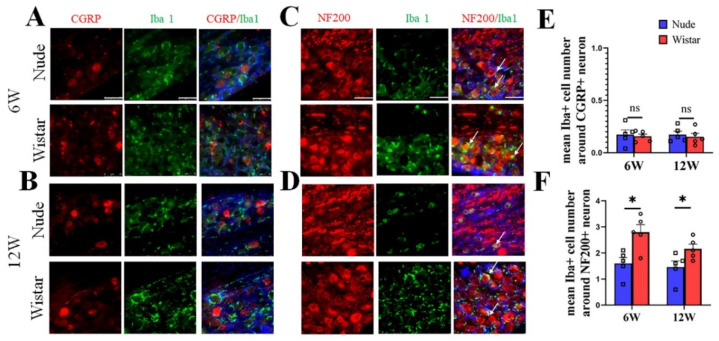
Immunofluorescence staining of Iba1 and CGRP/NF200 co-staining in DRG tissue. (**A**,**B**) Representative figures of CGRP (red) and Iba1 (green) co-staining in DRG tissue at 6 (**A**) or 12 weeks (**B**). (**C**,**D**) Representative figures of NF200 (red) and Iba1 (green) co-staining in DRG tissue at 6 (**C**) or 12 weeks (**D**). At 6 or 12 weeks in two groups, colocalization showed that Iba1 cells (white arrow) surround NF200+ neurons, but not CGRP+ neurons. (**E**) Quantitative analysis showed no difference in the mean number of Iba1+ cells around CGRP+ neurons in the DRG between the two groups. (**F**) Quantitative analysis showed the mean number of Iba1+ cells around NF200+ neurons in DRG of Wistar rats was higher than in nude rats both at 6 and 12 weeks. Scale bar 50 μm. Student’s *t*-test in (**E**,**F**). Sample size (n): n = 5. * *p* < 0.05; nude rats vs. Wistar rats.

To verify the neuron type in autotomy following PNI further, NF200/CGRP and Iba1 in DRG tissue were co-stained. The DRG contains neurons, satellite cells, and macrophages. Iba1 is a specific marker for activated macrophages or microglia and is used to label macrophages in the DRG, but not for satellite cells [6,28,29,30,31]. Our immunofluorescence staining studies showed that macrophages in the DRG surround relatively larger neurons, which is similar to previous reports [6,30]. In Wistar rats, few Iba1-marked cells were found around CGRP-marked neurons, while most Iba1-marked cells were primarily found around NF200-marked neurons at 6 weeks (Figure 4A). A similar phenomenon was observed at 12 weeks in the Wistar rats (Figure 4B). The same outcome was observed in nude rats at 6 and 12 weeks (Figure 4A,B). A quantitative analysis showed no difference in the mean number of Iba1+ cells around CGRP+ neurons in the DRG between the two groups at 6 (0.15 ± 0.03 vs. 0.17 ± 0.03, *p* > 0.05) and 12 (0.16 ± 0.02 vs. 0.17 ± 0.04, *p* > 0.05) weeks. The mean number of Iba1+ cells around the NF200+ neuron in the DRG of Wistar rat was higher than nude rats both at 6 (2.8 ± 0.29 vs. 1.6 ± 0.22, *p* < 0.05) and 12 (2.2 ± 0.18 vs. 1.5 ± 0.24, *p* < 0.05) weeks. Co-labeling of M1 (iNOS) or M2 (CD206) cells with NF200 identified that both M1 and M2 positive cells were predominantly located around NF200-positive neurons at 6 and 12 weeks (Supplementary Figure S2). These results suggest that NF200-marked neurons coexist with increased activated macrophages and myelinated A fiber neurons in rats developing autotomy.

### 2.5. CD4+ T Cells in the DRG at 6 and 12 Weeks

**Figure 5:**
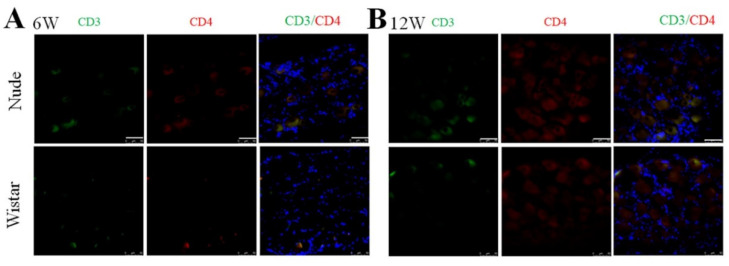
Immunofluorescence staining of CD3 and CD4 cells in DRG. At (**A**) 6 or (**B**) 12 weeks, CD3 and CD4+ cells were not found in either group. Scale bar 50 μm.

The nude rat is an immunodeficient type of rat characterized by a lack of appropriate development and education of functional T-cell lymphocytes [32]. It was reported that the secretion of interferon-γ (INF- γ) by CD4+ T cells can promote the activation of macrophages [33,34]. Thus, we examined the presence of CD4+ T cells (CD3 and CD4 marked) in the DRG in both groups to explore the origin of the autotomy difference and macrophage activation between the two groups further. Before PNI, no CD3+ or CD4+ cells in the DRG were found in nude and Wistar rats (Supplementary Figure S3), indicating that T cells were not present in normal DRG tissue. As expected, neither CD3+ nor CD4+ cells were found in the DRG of nude rats at 6 or 12 weeks. No infiltrated CD3+ or CD4+ T cells were found at 6 or 12 weeks in Wistar rats (Figure 5). The effectiveness of the CD3 and CD4 antibodies was verified by a positive control of the spleen from Wistar rats (Supplementary Figure S4).

## 3. Discussion

Autotomy is very common in animals following different injury types of the peripheral nerve, including crush, transection, and re-suturing [5,35]. Defects of the toe induced by self-mutilation affect the functional assessment of peripheral nerve regeneration [5,36]. Severe autotomy may also result in infection, poor mental health, and weight loss in animals, which creates animal welfare concerns. However, studies of autotomy in animals are limited. We have historically observed less autotomy behavior after PNI in nude rats compared to Wistar rats. The motivation of this work is to improve the design of experiments, such as animal and group size selection, for animal-model-based peripheral nerve regeneration studies and better understand the cellular behavior that is associated with the different behavioral states. This study is the first to compare the autotomy behavior following PNI between Wistar and nude rats in terms of incidence and severity. We also examined differences among the cellular behavior in each animal model associated with the behavioral states, which provides new insight into the cellular processes that drive the pain response following sciatic nerve transection.

Severe autotomy can require animals to be euthanized prior to the intended endpoint of studies, thereby leading to early endpoints that may reduce the significance of the experimental study (e.g., due to an absence of experimental data). This comparison study demonstrates that autotomy in Wistar rats occurs more frequently and exhibits higher scores relative to nude rats. After complete transection and repair of the sciatic nerve, the autotomy rate in Wistar rats was as high as ~80%, which is similar to a previous report [37]. Moreover, while autotomy was severe in Wistar rats (scores ≥ 4), nude rats exhibited less severe autotomy behavior. We also found that autotomy in Wistar rats typically developed into severe autotomy at the later stage, which affects functional assessment. While autotomy can be used as an effective evaluation index, the disadvantages of autotomy on research outcomes should also be considered for experiment design in animal-model-based PNI studies.

Understanding differences in cellular behavior between the two groups could identify key factors to help reduce and treat autotomy. While it is established that macrophages play a key role in neuropathic pain [12,38,39], differences in macrophages between nude and Wistar rats after PNI contributing to anatomy have yet to be reported. Though we did not find CD4+ T cells in the DRG in both groups at 6 or 12 weeks after PNI, relatively more activated macrophages and NF200+ neurons were found in the DRG of Wistar rats. Further, the activated macrophages were primarily found around NF200-marked myelinated A fiber neurons of the DRG in both Wistar and nude rats, suggesting a potential cellular interaction between myelinated A fiber neurons and activated macrophages, which might be a significant contributor to autotomy in rats.

While prior studies attribute autotomy behavior to the formation of neuromas [8,10,11], the mechanisms of macrophage activation and communication with neurons are not fully understood. Macrophages in the DRG were reported to contribute to both the initiation and persistence of neuropathic pain in wild mice, rather than the macrophages at the injury site [12]. Prior studies show that the number of activated macrophages (Iba1+ cells) in the DRG began increasing 2–4 days after peripheral nerve injury and remained elevated in early phases (2–4 weeks) [6,30,40,41]. However, macrophage behavior at or after 6 weeks and comparisons between Wistar and nude rats have yet to be reported. Thus, we selected the 6-week (rapid development period) and 12-week (slow development period) periods as the two endpoints for tissue analysis in this study. Our data, collected using a transection-repair model, revealed that macrophages accumulate in the DRG of Wistar and nude and remain present for more than 12 weeks. Consistent with autotomy behavior, there were more activated macrophages present in the DRG of Wistar rats than nude rats. However, at 12 weeks, activated macrophages in the DRG of Wistar rats were significantly reduced compared to 6 weeks. This may explain why autotomy developed at a slower rate throughout the 6–12-week interval. Combined with previous reports, our data suggest that activated macrophages in the DRG are associated with differences in autotomy behavior between Wistar and nude rats. Future studies may consider additional time points to elucidate further the temporal profile of activated (Iba1+) macrophages as well as to evaluate its critical role in sciatic nerve transection-induced autotomy and the mechanistic pathways underlying autotomy and its potential therapy.

Neurons in the DRG, as primary neurons in sensory pathways, play a key role in the occurrence and maintenance of pain [2,4]. Primary neurons are composed of heterogeneous subtypes, including myelinated A fibers and peptidergic C fibers [19]. Different subtypes of primary neurons have a distinct intrinsic growth capacity and show heterogeneous responses to nerve stimuli [42]. PNI leads to changes in the proportion of different neurons in the DRG, which is attributed to the unique responses and anti-apoptotic ability of different subtypes of neurons [42,43,44]. Macrophages influence the activation and apoptosis of neurons, which also leads to different proportions of NF200+ or CGRP+ neurons between the two groups [24,45]. We found that while the amount of CGRP+ neurons in the DRG did not differ between Wistar and nude rats, Wistar rats exhibited significantly more NF200+ neurons in the DRG at 6 and 12 weeks. NF200 is a marker of myelinated A fiber neurons, and CGRP is a marker of small/medium peptidergic neurons [46]. These data indicate that myelinated A fiber (NF200+) neurons may contribute to autotomy rather than small/medium peptidergic (CGRP+) neurons.

In addition to the differences in macrophage accumulation associated with the different behavioral states, we also observed differences in the number of NF200+ neurons and macrophage accumulation in the DRG among the two groups. The majority of activated macrophages were found around NF200-marked neurons in the DRG of both Wistar and nude rats. Peripheral sensitization was previously shown to play a critical role in the initiation and maintenance of pain [13,20,39]. Many studies demonstrate that macrophages in the DRG communicate with neurons and contribute to neuronal sensitivity [2,12,39,47,48]. Thus, there is a well-established interaction between immune cells and nerves following nerve injury, yet the understanding of the interaction is limited. In our study, more activated macrophages were present in the DRG of Wistar rats than nude rats, which is consistent with more autotomy behavior and higher scores in Wistar rats. In addition, the higher number of NF200+ neurons in Wistar rats further supports that the accumulation and activation of macrophages may account for the difference in autotomy between the two groups. These results also suggest that A fiber neurons in the DRG could play an important role in autotomy and neuropathic pain. For example, inhibiting macrophage activation or targeting A fiber neurons may provide a future strategy for treating autotomy and pain.

The nude rat was derived from the Foxn1 gene mutation in Wistar rats in 1953 [49] and is widely used in the study of immunity and tumor biology. In comparison with normal Wistar rats, the nude rat is an immunodeficient type of rat characterized by the lack of appropriate development and education of functional T-cell lymphocytes [32]. In vitro and in vivo studies revealed that the number, activity, and function of macrophages derived from Wistar and nude rats were similar [49,50,51]. While potential Foxn1-associated pain mechanisms have yet to be reported, we originally hypothesized that the difference in macrophages may be related to T cells, CD4+ cells, which are primary helper T cells. However, CD4+ cells were not found in the DRG of Wistar and nude rats at 6 and 12 weeks. Previous reports show that T cells are rarely found in normal DRG tissues [33,52]. CD4+ T cells are also shown to infiltrate the DRG in the early stages after nerve injury [33,52]. Similarly, CD4+ T cells can be found in the DRG at the onset of an experimental autoimmune encephalomyelitis (EAE) model and yet are absent at the chronic time point, suggesting that CD4+ cells exhibit transient expression in the DRG [52]. In our study, no CD4+ T cells were observed in the DRG of both groups, which suggests that CD4+ T cells do not contribute to autotomy or pain at late time points after nerve repair (6 or 12 weeks). It was reported that the secretion of INF- γ by CD4+ T cells can promote the activation of macrophages [33,34]. CD4+ T cells migrate to the DRG and secrete IFN-γ at 7 days after viral infection [53]. IFN-γ secreted by CD4+ T cells may be the main factor of excessive macrophage activation in Wistar rats. Future studies are needed with more early time points to evaluate temporal profiles and the differences of T cells including CD4+ T cells in these two groups. Other mechanism-focused studies including the deletion and supplement of activated macrophages in the DRG are an important aspect of understanding the mechanistic roles of macrophages in autotomy and may be considered in future research.

## 4. Materials and Methods

### 4.1. Animals and Surgery Procedure

All animals were maintained according to NIH guidelines, and experimental protocols were approved by the Institute Animal Care and Use Committee (IACUC) of the University of Maryland, Baltimore. All animals are maintained in good and similar conditions with clean cages changed 3 times a week. The repair model of sciatic nerve injury is consistent with our previous studies using 3D-printed nerve guidance channels [54]. In brief, 14 nude rats (Charles River, Wilmington, MA, USA, 7–8 weeks old) and 14 Wistar (Charles River, Wilmington, MA, USA, 7–8 weeks old) rats were randomly assigned. The animals were anesthetized with isoflurane, and the left sciatic nerve was exposed. Then, 15 mm of the nerve were removed and bridged with 3D-printed silicone nerve scaffolds filled with 2 × 10^6^ neural crest stem cells in a collagen gel. At 6 and 12 weeks after surgery, half of the rats in each group were euthanized, and tissues were harvested. Five sham Wistar rats and five sham nude rats were included as a control.

### 4.2. Autotomy Assessment

Rats were examined weekly after surgery-foot self-mutilation, and weight and activity status were recorded. Autotomy was scored with a modified Wall’s Score [5], in which a score of one was added for each affected digit, and a further score of one was assigned for each affected metatarsus. Since each nail represents a different autotomy intensity and coverage, an additional score of one was assigned for the deficiency of each nail. The maximum score for this scale was 15 points (Table 1, Figure 1A) [5,55]. Scores greater than four were considered severe. All animals were monitored for 12 weeks, and the average autotomy scores at 6 and 12 weeks after surgery were used for further analysis.

### 4.3. Tissue Preparation

At 6 and 12 weeks after surgery, half of the rats in each group selected at random were deeply anesthetized and transcardially perfused with phosphate buffer (PBS, 0.16 M, pH 7.2, 37 °C) and paraformaldehyde (PFA, 4%, 37 °C). After perfusion, bilateral lumbar (L)-4/5 DRG and sciatic nerves were harvested and fixed overnight with 4% PFA in PBS. The tissues were then treated with 30% sucrose and embedded with an optimal cutting temperature compound (Fisher Health Care, Houston, TX, USA) for cryopreservation. Sections (10 μm) were cut in a cryostat and mounted to slides.

### 4.4. Immunofluorescence Analysis

Standard immunofluorescence analyses were performed as we previously described [15,56] using a fluorescence microscope (Leica, DMi8, Germany). Monoclonal antibodies against the following proteins were used: Iba-1 (activated macrophages marker, 1:300, Abcam, Waltham, MA, USA), iNOS (M1 macrophages marker, 1:500, Abcam, Waltham, MA, USA), CD206 (M2 macrophages marker, 1:500, Abcam, Waltham, MA, USA), CGRP (small/medium peptidergic neurons marker, 1:100, Abcam, Waltham, MA, USA), NF200 (myelinated A fiber neurons marker, 1:500, Abcam, Waltham, MA, USA), CD4 (CD4+ T cells marker, 1:100, Abcam, Waltham, MA, USA). Secondary antibodies against the primary antibody were used: goat anti-rabbit IgG antibodies conjugated with Alexa 488 (1:500, Invitrogen, Waltham, MA, USA), goat anti-rabbit IgG antibodies conjugated with Alexa 568 (1:500, Invitrogen, Waltham, MA, USA), monkey anti-goat IgG antibodies conjugated with Alexa 488 (1:500, Invitrogen, Waltham, MA, USA), monkey anti-mouse IgG antibodies conjugated with Alexa 568 (1:500, Invitrogen, Waltham, MA, USA).

### 4.5. Quantitative Image Analysis

For image analysis, data were collected from at least three sections of DRG tissue for each rat. At a minimum, three randomized microscopic fields (50 μm × 50 μm) were captured in one section, and averages were used for further analysis. Five of seven rats were randomly selected from each group at each point time for immunofluorescence staining. The number of positive cells in the images was blindly counted using Image J software (National Institutes of Health, Bethesda, MD, USA) [15] and averaged for further analysis. To determine the percentage of labeled neurons in the DRG, the number of positive neurons (three times the background signal) was divided by the total number of neurons [6].

### 4.6. Statistical Analysis

Data are expressed as mean ± standard error of the mean (SEM). All data followed a normal distribution. The significance among differences between mean values was determined by Student *t*-tests for two groups or two-way ANOVA with Bonferroni’s post-test as indicated for multiple groups. The statistical analysis was performed using statistical software (SPSS 20.0, IBM/SPSS Inc., Chicago, IL, USA).

## 5. Conclusions

**Figure 6:**
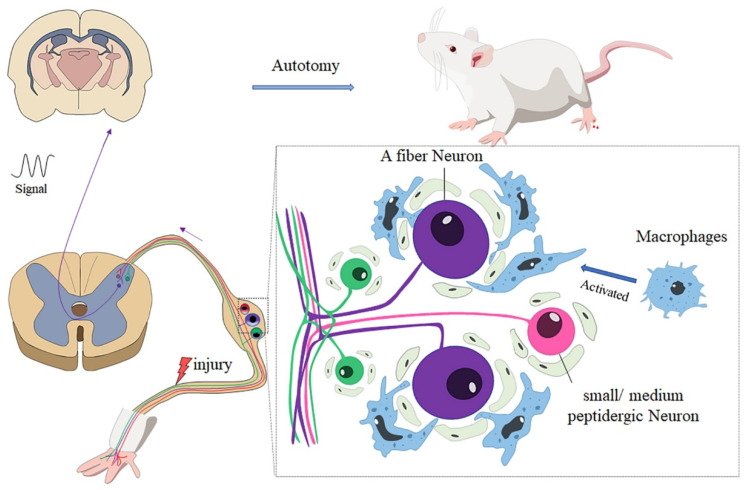
After peripheral nerve injury, macrophages in DRG are activated and distributed around A sensory neurons; the neuro-immunity between macrophages and neurons enhances the generation of abnormal signals and transmits the signal to the brain, which may contribute to autotomy behavior.

In summary, our research showed that there was less autotomy in nude rats than Wistar rats. Further analysis revealed that the accumulation and activation of macrophages may account for the difference in autotomy between the two groups. Immunofluorescence studies revealed that A neurons in the DRG play an important role in autotomy and neuropathic pain (Figure 6).

## Figures and Tables

**Table 1:** The modified Wall’s Score.

Mutilation	Score
No mutilation	0
Nail mutilation (one finger)	1
Phalange mutilation (one finger)	2
Metacarpal mutilation (one finger)	3

## Data Availability

This study did not generate any unique datasets. Please contact the lead contact for additional information.

## Supplementary Materials

The following are available online at https://www.mdpi.com/article/10.3390/ijms222312801/s1.
