# Supplementary material for: Macrophage Activation in the Dorsal Root Ganglion in Rats Developing Autotomy after Peripheral Nerve Injury
Source: Int J Mol Sci. 2021 Nov 26;22(23):12801. doi: 10.3390/ijms222312801 (PMC8657625; doi:10.3390/ijms222312801)
Supplement: Supplementary file 1 [file ijms-22-12801-s001.zip › ijms-1365442-supplementary.pdf]

International Journal of *Molecular Sciences*

MDPI

Macrophage Activation in the Dorsal Root Ganglion in Rats Developing Autotomy after Peripheral Nerve Injury

Xiang Xu [1], Xijie Zhou [1], Jian Du [1], Xiao Liu [1], Liming Qing [1], Blake N. Johnson [2] and Xiaofeng Jia [1,3,4,5,6,*]

1 Department of Neurosurgery, University of Maryland School of Medicine, Baltimore, MD 21201, USA; Xiang.xu@som.umaryland.edu (X.X.); xzhou@som.umaryland.edu (X.Z.); JDu@som.umaryland.edu (J.D.); xiao.liu@som.umaryland.edu (X.L.); LQing@som.umaryland.edu (L.Q.)

2 Department of Industrial and Systems Engineering, School of Neuroscience, Virginia Tech, Blacksburg, VA 24061, USA; bnj@vt.edu

3 Department of Biomedical Engineering, Johns Hopkins University School of Medicine, Baltimore, MD 21205, USA

4 Department of Anesthesiology and Critical Care Medicine, Johns Hopkins University School of Medicine, Baltimore, MD 21205, USA

5 Department of Orthopedics, University of Maryland School of Medicine, Baltimore, MD 21201, USA

6 Department of Anatomy and Neurobiology, University of Maryland School of Medicine, Baltimore, MD 21201, USA

* Correspondence: xjia@som.umaryland.edu; Tel.: +1-410-706-5026

Citation: Xu, X.; Zhou, X.; Du, J.; Liu, X.; Qing, L.; Johnson, B.N.; Jia, X. Macrophage Activation in the Dorsal Root Ganglion in Rats Developing Autotomy after Peripheral Nerve Injury. *Int. J. Mol. Sci.* **2021**, *22*, 12801. https://doi.org/10.3390/ijms222312801

Academic Editors: Mike Barbeck and Yoshiro Kobayashi

Received: 18 August 2021
Accepted: 18 November 2021
Published: 26 November 2021

Publisher's Note: MDPI stays neutral with regard to jurisdictional claims in published maps and institutional affiliations.

Copyright: © 2021 by the authors. Licensee MDPI, Basel, Switzerland. This article is an open access article distributed under the terms and conditions of the Creative Commons Attribution (CC BY) license (https://creativecommons.org/licenses/by/4.0/).

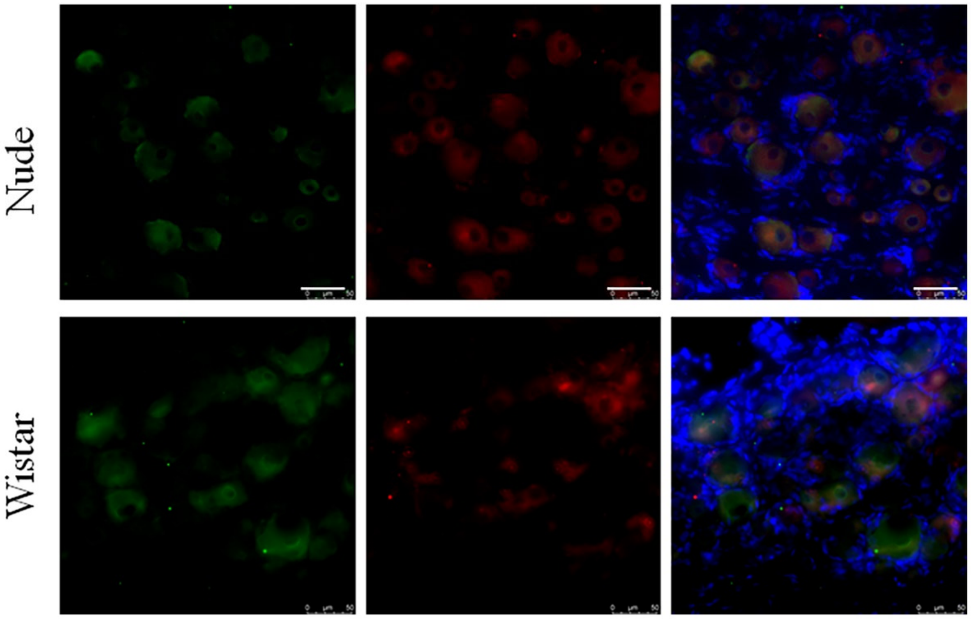

Supplementary Fig S1. Immunofluorescence staining of negative control in DRG tissue. Green, monkey anti-goat IgG antibodies conjugated with Alexa 488. Red, monkey anti-mouse IgG antibodies conjugated with Alexa 568. Scale bar = 50 μm.

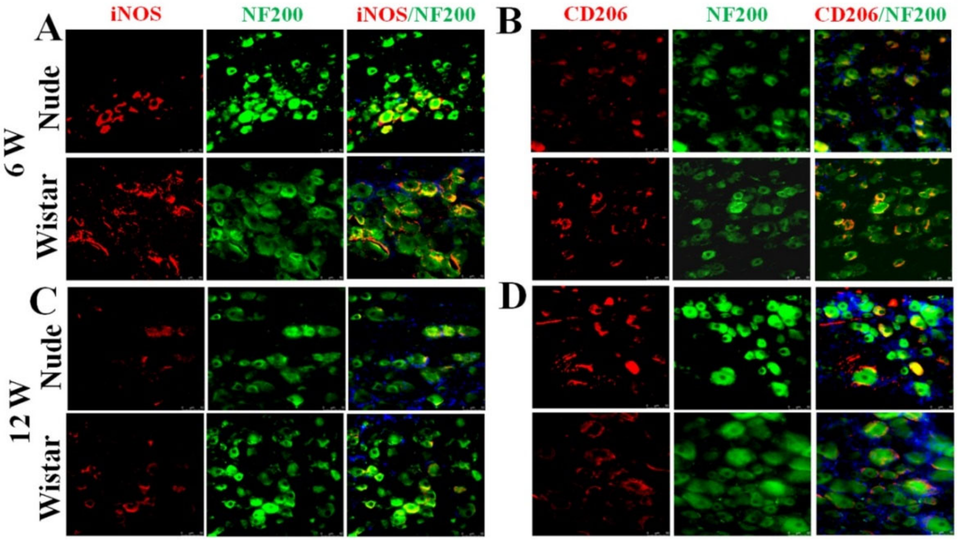

Supplementary Fig S2. Immunofluorescence staining of M1 (iNOS) and M2(CD206) with NF200 co-staining in DRG tissue. Representative iNOS (red) and NF200 (green) co-staining in DRG tissue at 6 weeks (A) or 12 weeks (C). Representative CD206 (red) and NF200 (green) co-staining in DRG tissue at 6 weeks (B) or 12 weeks (D). Scale bar = 50 μm.

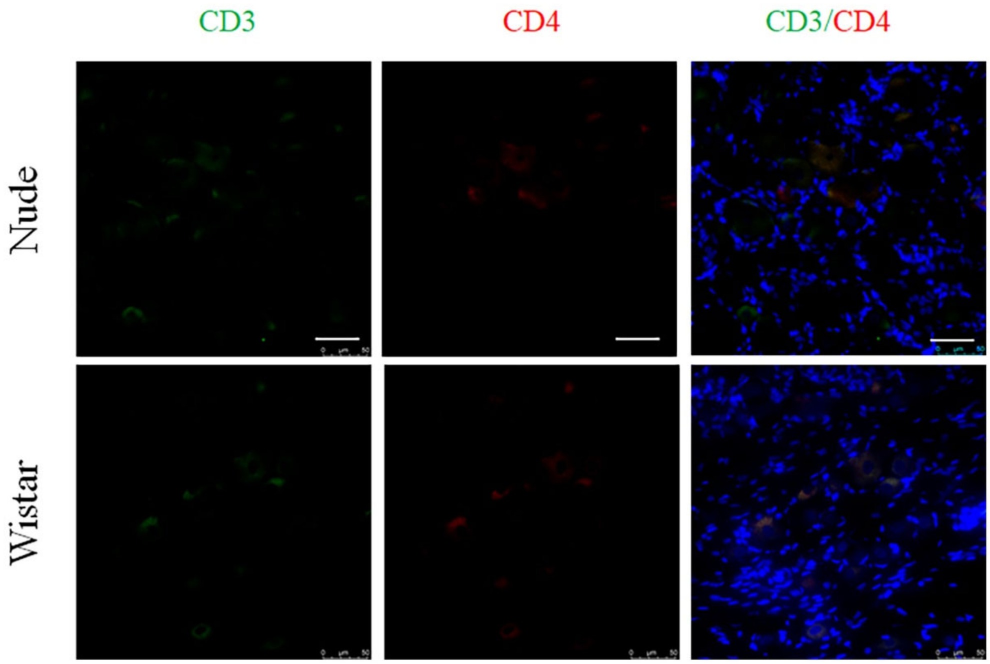

Supplementary Fig S3. Immunofluorescence staining of T cells in DRG tissue before PNI. Representative CD4 (red) and CD3 (green) co-staining in DRG tissue in nude rats or Wistar rats before peripheral nerve injury (PNI). Scale bar = 50 μm.

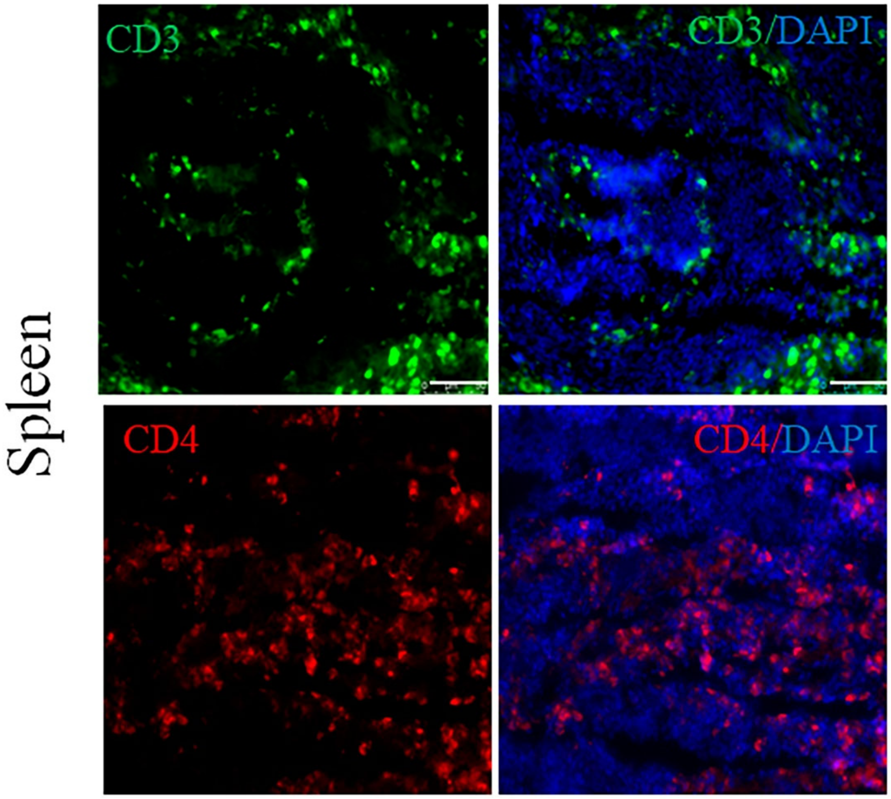

Supplementary Fig S4. Immunofluorescence staining of CD3+ T cells (green) and CD4+ T cells (red) positive control in Spleen tissue. Scale bar = 50 μm.
